# When the Unexpected Happens: Diffuse Alveolar Hemorrhage in Negative-Pressure Pulmonary Edema

**DOI:** 10.7759/cureus.87323

**Published:** 2025-07-05

**Authors:** David Costa, Luís Teles, Nuno Prucha Leite, José Tadeu

**Affiliations:** 1 Intensive Care Medicine Department, Unidade Local de Saúde de Entre Douro e Vouga, Santa Maria da Feira, PRT

**Keywords:** acute airway obstruction, congenital hydrocephalus, diffuse alveolar hemorrhage, laryngospasm, negative-pressure pulmonary edema, pulmonary critical care, pulmonary edema, seizure

## Abstract

Negative-pressure pulmonary edema (NPPE) is an uncommon complication of acute airway obstruction, classically resulting in non-cardiogenic pulmonary edema. Diffuse alveolar hemorrhage (DAH) occurring in the setting of NPPE is exceedingly rare but can be life-threatening. We present the case of a young adult who developed NPPE complicated by DAH following a brief episode of acute upper airway obstruction. The patient exhibited sudden hemoptysis and respiratory failure, with imaging confirming pulmonary edema and new bilateral infiltrates consistent with hemorrhage. Bronchoscopic evaluation and laboratory workup excluded other causes of DAH. Supportive management, including airway protection, mechanical ventilation with positive end-expiratory pressure, and careful hemodynamic support, led to rapid clinical improvement. The patient made a full recovery with complete resolution of pulmonary infiltrates. This case underscores that even short-lived airway obstruction can precipitate DAH via NPPE, highlighting the importance of prompt recognition and management of this unexpected complication.

## Introduction

Negative-pressure pulmonary edema (NPPE) is a rare condition (occurring in up to 0.1% of patients undergoing orotracheal intubation), and a type of noncardiogenic pulmonary edema that results from a severe inspiratory effort against an obstructed airway. It most commonly arises in situations such as laryngospasm, choking, or other forms of upper airway blockage, where the patient generates high negative intrathoracic pressure in an attempt to inhale against the obstruction. This severe negative pressure causes a rapid shift of fluid into the alveoli, leading to pulmonary edema. NPPE typically presents immediately or within minutes after the obstruction, often in young, otherwise healthy individuals, and it is generally reversible with timely supportive care [1]. Documented causes of NPPE include strangulation, epiglottitis, thyroid goiter, obstructive sleep apnea, endotracheal intubation biting, foreign body inhalation, and generalized tonic-clonic seizure [2].

The NPPE can lead to diffuse alveolar hemorrhage (DAH), defined by bleeding into the alveolar spaces, accounting for only 5% of the etiologies leading to DAH. Clinically, DAH may manifest as hemoptysis, dropping hemoglobin levels, and diffuse pulmonary infiltrates with hypoxemic respiratory failure. The causes of DAH are diverse (including immune-mediated vasculitides, coagulopathies, infections, and toxins), but in the perioperative or immediate post-extubation setting, DAH is most frequently reported in association with NPPE due to acute airway obstruction [2,3].

We report a case of NPPE-associated diffuse alveolar hemorrhage in an adult patient following a transient upper airway obstruction. This case highlights the need to consider DAH in any patient with NPPE who presents with hemoptysis, and it underscores the importance of rapid diagnosis and management to ensure a favorable outcome.

## Case presentation

A 25-year-old previously healthy male individual (American Society of Anesthesiologists physical status classification system I) underwent an urgent laparoscopic appendectomy and was given balanced general anesthesia using propofol, fentanyl, lidocaine, and rocuronium, with maintenance provided by desflurane. The intubation was routine, and the surgical procedure was uncomplicated. Upon awakening from anesthesia, immediately after extubation in the operating room, the patient experienced an acute upper airway obstruction characterized by laryngospasm. He developed inspiratory stridor and was unable to ventilate, with oxygen saturation rapidly dropping to 70%. Anesthesia staff promptly intervened with 100% oxygen and positive-pressure ventilation via face mask (Boussignac mask at 5 cm H_2_O) and Larson's maneuver.

Soon after the laryngospasm episode, the patient began experiencing moderate hemoptysis, leading the medical team to administer tranexamic acid via bolus and infusion. Despite these efforts, the patient's condition deteriorated over several minutes, with a PaO_2_/FiO_2_ ratio of 62, necessitating an emergent orotracheal intubation.

**Figure 1:**
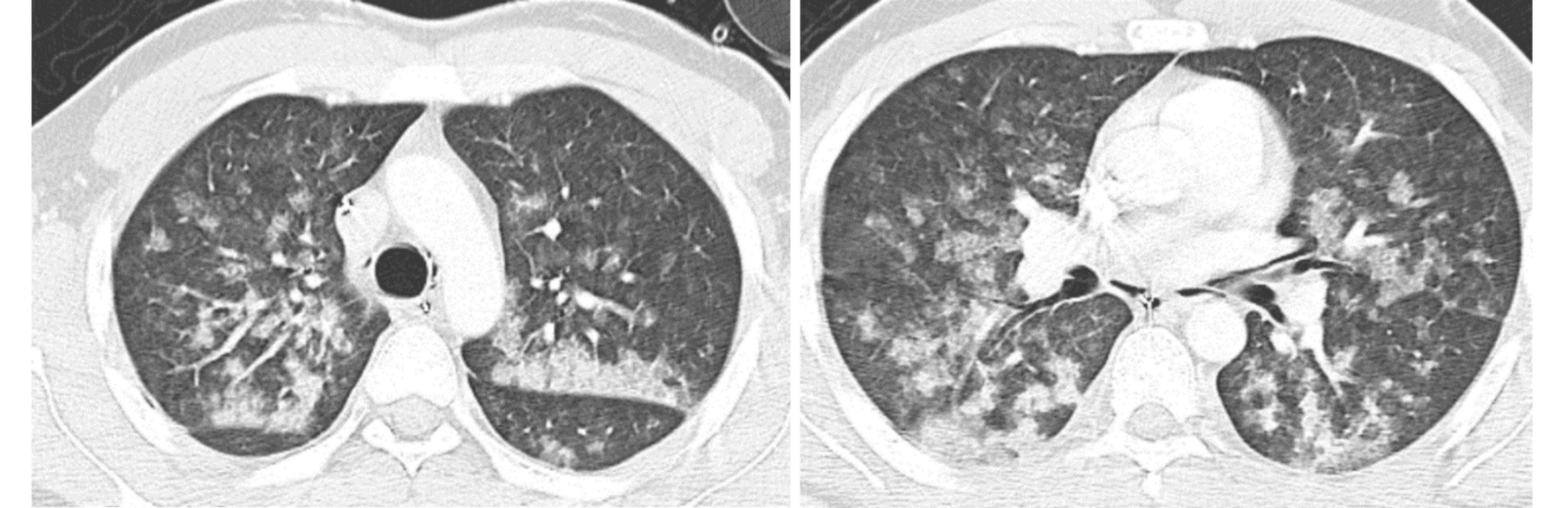
CT scan of the thorax showing alveolar hemorrhage

Upon re-intubation, frothy pink secretions were noted in the endotracheal tube, and the patient began coughing up blood-tinged fluid consistent with hemoptysis. The patient remained hypoxemic (SpO_2_ in the mid-80% range on 100% O_2_) and was placed on mechanical ventilation. A chest CT scan at that time (Figure 1) revealed bilateral diffuse infiltrates, especially in perihilar regions, consistent with pulmonary edema. Given the clinical context, NPPE was diagnosed. However, the presence of frank blood in the airway raised concern for diffuse alveolar hemorrhage. The patient was transferred to the intensive care unit (ICU) for further management.

In the ICU, bronchoscopy was performed within a few hours of the event to evaluate the source of bleeding. Bronchoscopic examination showed diffuse bloody secretions throughout the bronchial tree with no focal source of bleeding, supporting the diagnosis of diffuse alveolar hemorrhage secondary to NPPE. During bronchoalveolar lavage, the retrieved fluid became progressively more hemorrhagic with each aliquot, a hallmark feature of diffuse alveolar hemorrhage. Laboratory investigations were unremarkable: coagulation studies were within normal limits, and autoimmune screening (including tests for antinuclear antibody, antineutrophil cytoplasmic antibody, and anti-glomerular basement membrane antibody) was negative. Cardiogenic pulmonary edema was ruled out by normal cardiac enzymes and an echocardiogram showing normal left ventricular function. There was no evidence of aspiration of blood from the surgical site, as the procedure was remote from the airway, and the bronchoscopy did not show any clots or bleeding originating from the upper airway or bronchi.

**Figure 2:**
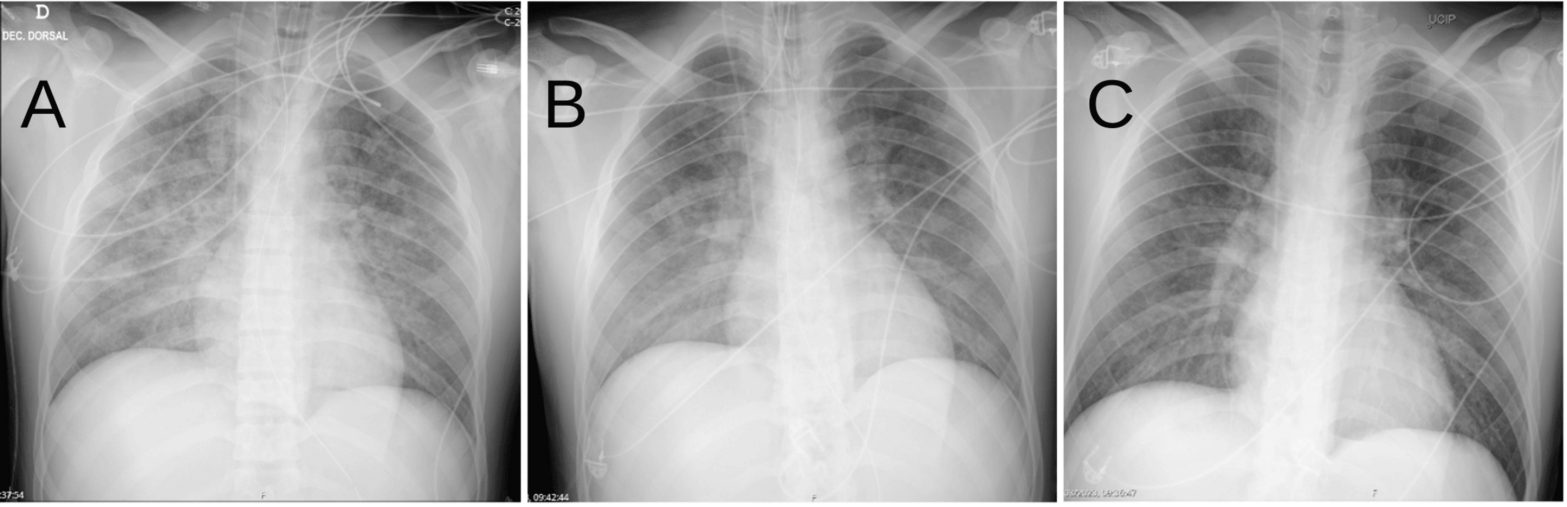
Evolution of chest X-ray at day one (A), day two (B), and day three (C) of intensive care unit stay

The patient was managed with supportive care. He remained on mechanical ventilation (with lung protective ventilation - plateau pressure of 30 cm H_2_O with a driving pressure of 15 cm H_2_O) with moderate positive end-expiratory pressure (15 cm H_2_O) to mitigate pulmonary edema and aid in alveolar recruitment. Diuretic therapy (intravenous furosemide at a dose of 60 mg/day) was administered to help fluid shift out of the lungs. Intravenous corticosteroids were given empirically for 48 hours to potentially reduce inflammation from alveolar injury, although their benefit in NPPE-related DAH is uncertain. The patient showed steady improvement over the next 24-36 hours. His oxygenation stabilized, and the volume of bloody secretions progressively decreased and then ceased. A repeat chest X-ray 24 and 48 hours later showed significant clearing of the infiltrates (Figure 2). The patient was extubated successfully on hospital day 2 and maintained normal oxygenation on room air. He was monitored for an additional day and then discharged without any residual respiratory symptoms. At a follow-up visit two weeks later, the patient was in good health, and a chest radiograph had returned to normal.

## Discussion

The patient in question was a 25-year-old male individual who underwent elective surgery under general anesthesia. Unfortunately, after the procedure, he experienced severe hypoxemic respiratory failure after extubation. Upon further investigation, it was determined that he had suffered from acute upper airway obstruction due to laryngospasm.

Laryngospasm occurs when the vocal cords spasm and close off the airway. This can happen due to a variety of reasons, including irritation, inflammation, or a reaction to medication. In this case, it was thought to be caused by the presence of excess soft tissue in the pharynx and muscle tone loss in the post-anesthetic state [4].

The diagnosis of postoperative NPPE is based on the precipitating factors, symptomatology, and chest radiograph. Precipitating factors for postoperative laryngospasm include anesthesia-related factors (such as airway irritation caused by selected anesthetics and insufficient depth of anesthesia). The clinical signs include tachypnea, tachycardia, rales, and rhonchi. Chest imaging shows rapid bilateral changes consistent with pulmonary edema [5].

The patient's attempt to inhale forcefully against the blocked airway resulted in acute negative pressure pulmonary edema, which is a rare but serious condition that can occur in patients who experience laryngospasm. It has been reported that a forceful inspiration against such glottic obstruction could result in a maximum intrathoracic pressure of −140 cm H_2_O from a baseline of −4 cm H_2_O. This event then leads to an increase in venous return and blood flow to the right side of the heart as well as a decrease in the flow from the left side as a result of increased afterload. This combination causes increased pulmonary blood volume and elevated pulmonary venous pressures, which lead to an increase in hydrostatic pressures and edema formation [6].

The patient also developed a frank alveolar hemorrhage. The exact cause of bleeding in NPPE is not entirely clear, but it is thought to be related to elevated stress on the pulmonary capillary walls. This stress can cause the mechanical disruption of the alveolar-capillary membrane, which impairs barrier function and leads to the leakage of blood into the air sacs. NPPE is a critical condition that can pose a life-threatening risk during the perioperative period if not promptly diagnosed and treated. It is crucial to identify potential causes, make a quick differential diagnosis, and determine an effective treatment to prevent disease progression [2,6].

According to the retrospective series by Contou et al., the DAH triad (hemoptysis, anemia, and pulmonary infiltrates) is observed in 50% of episodes, and acute respiratory failure in 94% [3]. Chest computed tomography reveals diffuse bilateral ground glass opacities in 100% of patients, while bronchoscopy detected bilateral hemorrhage and macroscopically bloody bronchoalveolar lavage, with siderophage absence in most, indicating acute DAH [3].

The management of NPPE is primarily supportive, focusing on early recognition and clearing of the upper airway, supplemental oxygen therapy, and the use of noninvasive ventilation, including continuous positive airway pressure (CPAP). In severe cases (such as the one described), re-intubation with positive end-expiratory pressure (PEEP) may be necessary. Low doses of succinylcholine can be used to alleviate laryngospasm. The effectiveness of steroids and diuretics in treating NPPE remains unclear (some clinicians administer corticosteroids in hemorrhagic cases to mitigate inflammatory lung injury, though robust evidence for steroids in NPPE or DAH is lacking), and there are no established recommendations for their use in this condition [7].

This case also highlights the importance of considering diffuse alveolar hemorrhage in the differential diagnosis of any hemoptysis that occurs in the immediate postoperative period or after an acute airway obstruction. Initial management should focus on stabilizing the patient, but diagnostic steps such as bronchoscopy and appropriate laboratory tests are crucial to confirm DAH and exclude other causes (for instance, anti-glomerular basement membrane disease or systemic vasculitis in a patient with pulmonary hemorrhage). In our patient, bronchoscopy was invaluable in demonstrating the diffuse nature of the bleeding and excluding focal lesions or significant aspiration of blood from the surgical site. Likewise, the normal cardiac evaluation helped rule out cardiogenic edema, and negative autoimmune tests helped exclude an underlying vasculitic process. By confirming the diagnosis of NPPE-related DAH, we were able to avoid more invasive investigations or immunosuppressive therapies that would be indicated for other causes of DAH.

## Conclusions

This case underscores the potential risks associated with standard surgery under general anesthesia, particularly during the perioperative period. While complications such as laryngospasm and NPPE are rare, they can have severe consequences if not promptly recognized and managed. The primary approach to treating NPPE is centered around supportive care. This involves addressing the root cause of airway obstruction, maintaining sufficient oxygenation, and, in severe cases, employing mechanical ventilation as necessary. In this case, the patient's acute negative pressure pulmonary edema and frank alveolar hemorrhage highlight the need for careful monitoring and management of respiratory function during the post-anesthetic period. Prompt diagnosis and treatment of NPPE are essential to prevent disease progression and reduce the risk of life-threatening complications. This requires a thorough understanding of the potential causes and risk factors associated with this condition, as well as a prompt and accurate differential diagnosis.

Overall, this case emphasizes the need for careful monitoring and management of patients undergoing surgery under general anesthesia. Healthcare professionals must remain vigilant for potential complications, be prepared to act quickly and decisively when they arise and work collaboratively to ensure the best possible outcomes for patients.
